# Seafood-Processing Sludge Composting: Changes to Microbial Communities and Physico-Chemical Parameters of Static Treatment versus for Turning during the Maturation Stage

**DOI:** 10.1371/journal.pone.0168590

**Published:** 2016-12-21

**Authors:** Iria Villar, David Alves, Salustiano Mato

**Affiliations:** Department of Ecology and Animal Biology, University of Vigo, Vigo, Pontevedra, Spain; Natural Environment Research Council, UNITED KINGDOM

## Abstract

In general, in composting facilities the active, or intensive, stage of the process is done separately from the maturation stage, using a specific technology and time. The pre-composted material to be matured can contain enough biodegradable substrates to cause microbial proliferation, which in turn can cause temperatures to increase. Therefore, not controlling the maturation period during waste management at an industrial level can result in undesired outcomes. The main hypothesis of this study is that controlling the maturation stage through turning provides one with an optimized process when compared to the static approach. The waste used was sludge from a seafood-processing plant, mixed with shredded wood (1:2, v/v). The composting system consists of an intensive stage in a 600L static reactor, followed by maturation in triplicate in 200L boxes for 112 days. Two tests were carried out with the same process in reactor and different treatments in boxes: static maturation and turning during maturation when the temperature went above 55°C. PLFAs, organic matter, pH, electrical conductivity, forms of nitrogen and carbon, hydrolytic enzymes and respiratory activity were periodically measured. Turning significantly increased the duration of the thermophilic phase and consequently increased the organic-matter degradation. PCA differentiated significantly the two treatments in function of tracking parameters, especially pH, total carbon, forms of nitrogen and C/N ratio. So, stability and maturity optimum values for compost were achieved in less time with turnings. Whereas turning resulted in microbial-group stabilization and a low mono/sat ratio, static treatment produced greater variability in microbial groups and a high mono/sat ratio, the presence of more degradable substrates causes changes in microbial communities and their study during maturation gives an approach of the state of organic-matter degradation. Obtaining quality compost and optimizing the composting process requires using turning as a control mechanism during maturation.

## Introduction

Food-processing industries in the European Union generate a large quantity of waste-products, estimated to be 35 megatonnes per year; 60% of this consists of organic material [1]. The seafood-processing industry generates effluent with a high content of fish remains and oils, which means wastewater with a high organic load [2]. For the management of organic sludge from wastewater treatment, one of the most widely-used techniques is composting. Composting is a process of biologically degrading solid organic substrates under aerobic conditions through the action of diverse microbial populations; through this process one obtains a stable-humified-product that is suitable for land application as a soil-improver and source of organic matter and nutrients [3]. The elevated availability of nourishment in organic waste causes microbial growth and with it an elevation in temperature and consequently the succession of different microbial communities which appear at different stages of the composting process. In the first phase, known as the mesophilic phase, proliferate mesophiles decompose the most basic organic compounds, causing the temperature to exceed 45°C. This increase in temperature results in the growth of thermophilic organisms and the inhibition of thermally-intolerant ones in what is known as the thermophilic phase. The final phases consist of a cooling period, which is characterized by the growth of mesophilic organisms, and a maturation period during which the organic material is stabilized and turned into humus, obtaining a product suitable for use as a soil amendment [4].

Generally speaking, industrial composting facilities differ in two different areas: the first one where the material undergoes intensive decomposition (corresponding to the initial mesophilic and thermophilic phases) and the second one where stabilization and maturation phases should take place. The intensive stage is characterized by high temperatures, elevated oxygen consumption, and the production of gaseous and liquid emissions [5] and, therefore, composting facilities pay special attention to this part of the process, carried out with a specific technique or technology, with greater control and tracking than the second one. When this stage is thought to be finished, or the established time of the intensive process ends, the pre-composted material is deposited in the maturation area where the degradation and polymerization of organic substances under mesophilic conditions should continue. Nevertheless, the material obtained after the intensive stage may not be sufficiently decomposed and may undergo reactivation during the maturation stage. For example, in facilities with composting tunnels, the most active phase is often limited to a specific time (a two-to-three week stay) after which the content removed from the tunnels is deposited in piles to mature [6]. The limited duration and the lack of mixture of the waste during the time in the tunnel usually cause the reactivation of the material in the maturation area and the amount of attention given to this phase depends on the composting facility, but typically the maturation area is simply considered to be a storage space for compost [7]. The lack of control during this phase can cause environmental problems such as smells and leachates; it can also lower the quality of the compost. In order to obtain stable and matured compost, composting-facility operation and design should integrate both phases [5]. In many cases it is not possible to make changes *a posteriori* on the technique used in the intensive stage but it is possible to act on the pre-composted material disposed in the maturation area. So, it is important to know how the management and control of the material deposited in maturation conditions similar to composting facilities affects the physical-chemical and biological parameters and if this management allows a reduction of time and/or quality improvement of compost.

Turning is usually employed as a means to aerate, homogenize, and control the temperature during composting [8]. The effects of turning, along with how often it occurs, have been studied by a wide range of authors, who have observed changes to physical, chemical, and biological characteristics which affect the rate of decomposition and consequently the time needed to achieve maturity and high-quality compost [9–12]. Nevertheless, information about the possible effects of dynamic or static management of the pre-composted waste has not been found; this is due to the fact that turning is generally carried out during the most active part of the process, or scheduled throughout the entire process.

Therefore, the intention of this study is to understand, at a pilot scale, the maturation process carried out at composting facilities, examining the effects of both static management and turning on temperature control during this stage of the process. Furthermore, there are few studies related to seafood-processing sludge composting and even fewer dealing with the maturation phase. In a previous experiment, Villar et al. [13] studied the maturation of this type of waste product in comparison with pig manure and sludge from an urban wastewater treatment plant. The research demonstrates that the seafood-processing sludge used contains a high level of biodegradable substrates, which cause it to reactivate when left to mature after an intensive period in a static reactor. This made it ideal to be used in this study.

The objectives of this research were, firstly, to study whether static management or turning for temperature control influence the physico-chemical and biological parameters and the microbial-community structure throughout the maturation phase and, secondly, to determine if controlling the maturation phase produced a more stable product in less time. This study addresses the hypothesis that controlling by turning the pre-composted sludge allows for an improvement in compost quality, a reduction in the time process to obtain appropriate stability and maturity parameters and a microbial-community profile different to the static treatment one.

## Materials and Methods

### Composting experiment

#### Composting substrates

For this experiment, the wastewater sludge was from a purifier from a plant that produces pre-cooked and flash-frozen fish and cephalopods; it was obtained after fats had been separated from the sludge and the waste product had been treated with coagulants and flocculants; pinewood shred to smaller than 3cm was used as a bulking agent. The initial characteristics of these materials can be seen in Table 1. Both materials were mixed in a volumetric ratio 1:2, respectively, as in the previous study [13].

**Table 1:** Physico-chemical composition of the materials used in the composting experiments.

	Seafood sludge	Bulking agent
**Moisture content (%)**	61.8 ± 0.4	41.4 ± 0.2
**Organic matter (% dw)**	87.7 ± 0.3	93.9 ± 0.4
**pH**	4.90 ± 0.04	6.66 ± 0.01
**Electrical conductivity (mS cm**^**-1**^**)**	0.55 ± 0.00	0.29 ± 0.01
**Total carbon (mg g**^**-1**^ **dw)**	513.7 ± 3.2	558.2 ± 2.8
**Total nitrogen (mg g**^**-1**^ **dw)**	19.24 ± 0.16	12.80 ± 0.29
**C/N ratio**	26.7 ± 0.2	43.6 ± 0.5
**Fat content (% dw)**	19.8 ± 0.8	N.D.

dw: dry weight, N.D: not detected

#### Composting design

Composting was carried out in two stages, the objective being to simulate waste-product management as seen in industrial composting facilities. The composting system has been described in detail in Villar et al. [13]. In brief, the sludge-and-bulking-agent mixture was composted in a 600-liter-capacity static reactor; aeration was achieved through programmed forced ventilation of 400 m^3^/h by a periodic flow rate of 30 seconds every 90 min and an alarm flow for temperature and oxygen control. The material was kept in the reactor, where it underwent the intensive stage, until the temperature had dropped to below 35°C. The reactor was emptied and the homogenized material placed in triplicate in 200-liter-capacity wooden boxes where the cooling, stabilization, and maturation stage took place (henceforth called the maturation phase). The top parts of these boxes were open, their bottom part perforated; inside them, the material rested between two layers of bulking agent to lend some thermal insulation to the pre-composted material.

Two composting tests were carried out, using the same mixture and handling in the reactor; each test, however, received a different treatment in boxes, either static treatment or turning treatment for temperature control. The boxes that received the static treatment were left for 112 days without being homogenized, whereas the ones that received the turning treatment were mixed when temperatures went above 55°C to aerate, avoid excess temperature and homogenize the compost. The turnings were carried out in order to simulate industrial-level compost turning by emptying completely each box, thoroughly mixing and placing it again inside the box. The compost was moistened when moisture levels fell below 40% during both types of treatment. The boxes underwent oxygen and temperature control daily for the first 42 days and three times a week afterwards until the end of the 112-day process. 1500-milliliter of composite samples were taken from each box on day 0 (when the reactor was emptied) and on the 14th, 28th, 42nd, 56th, 70th, and 91st days of being in the box; they were screened through a one-centimeter sieve in order to remove the bulking agent. Once 112 days had passed, the boxes were emptied and all the compost screened. The samples were divided in three parts. One part was dried for two days at 40°C and ground in order to determine the total amount of carbon and nitrogen. Another part was freeze-dried and ground using mortar-and-pestle in order to analyze phospholipid fatty acids (PLFAs). The remaining part was kept fresh for the rest of the analytical determinations.

### Composting parameters

Moisture and organic matter contents of samples were calculated gravimetrically after drying at 105°C until constant weight and combustion at 550°C for 4 h, respectively. Total carbon (TC) and total nitrogen (TN) contents were determined by combustion using a LECO 2000 CN elemental analyzer. Inorganic nitrogen (N-NH_4_^+^ and N-NO_3_^−^) was determined in 0.5 M K_2_SO_4_ extracts (1:10, w/v) using the modified indophenol blue colorimetric method [14]. Total extractable nitrogen was determined in the same extracts after oxidation with K_2_S_2_O_8_, as described by Cabrera and Beare [15], and dissolved organic nitrogen content (DON) was calculated as (total extractable N)–(inorganic N). Water soluble carbon content (WSC) was analyzed in aqueous extracts (1:5, w/v) by dichromate oxidation in sulfuric acid solution. Electrical conductivity and pH were determined in aqueous extracts (1:10, w/v) using a pH meter Crison Basic 20 and a conductivimeter Crison CM 35. Fat content was determined in samples from day 56 and 112 by Soxhlet method using n-hexane as organic solvent [16].

The microbial community composition and biomass were determined by PLFA analysis following the method described by Gómez-Brandón et al. [17] for organic samples. The analysis was performed with a CP-Select FAME, 100m x 0.25mm in a gas chromatograph-mass spectrometer (GC-MS). Identification was done by comparison of retention times and mass spectra with known external standards (Larodan Fine Chemicals AB, Malmo, Sweden) and quantification was performed with methyl nonadecanoate fatty acid (C19:0) as internal standard. PLFAs were used to estimate the biomass of specific microbial groups: Gram-positive bacteria (i14:0, i15:0, a15:0, i16:0, a17:0), Gram-negative bacteria (15:1ω5, 16:1ω7, 17:1ω7, 18:1ω7) and fungi (18:2ω6, 18:1ω9, 20:1ω9). The total amount of PLFAs identified (totPLFAs) was used as an indicator of the viable microbial biomass [18]. The ratio of monounsaturated PLFAs to saturated PLFAs (mono/sat) was used as an indicator of physiological or nutritional stress in microbial communities [19].

β-glucosidase was estimated by the colorimetric measurement of p-nitrophenol released after incubation 1 g of fresh sample with 1 mL of p-nitrophenyl-β-D-glucopiranoside (0.025 M) for 1 h at 37°C [20]. Alkaline phosphatase was measured by incubating 0.5 g of fresh sample with 1 mL of p-nitrophenylphosphate (0.015 M) for 1 h at 37°C and subsequent colorimetric measurement of p-nitrophenol released [21]. Protease activity was measured after the incubation of 1 g fresh sample with 5 mL of sodium caseinate (2%) for 2 h at 50°C and subsequent determination of the amino acids released using Folin-Ciocalteu reagent [22].

Static respiration rate (SR) was measured using manometric respirometers by OxiTop^®^ system (WTW GmbH, Weilheim, Germany). Briefly, fresh weight equivalent to 4 g of dry sample was placed in a hermetic container with a 1 M NaOH trap to capture CO_2_, the pressure drop, due to microbial oxygen consumption, was recorded during 24 h at constant temperature. Germination index (GI) was calculated on day 56 and 112 by determining seed germination and root length of *Lepidium sativum* growing in 2 mL of aqueous extracts (1:5, w/v) in Petri dishes lined with paper filter during 48 h [23]. The self-heating test was carried out in the final compost using 2 L Dewar flask for 10 days at room temperature.

### Statistical analysis

All statistical tests were performed using R software [24]. Principal component analysis (PCA) was performed on the correlation matrix after normalization to zero mean and variance unit of the variables. The PCA was conducted using the prcomp function and the package factoextra [25]. Mixed models were fitted with the nlme package [26] to evaluate the differences between treatments. The waste type and time were fixed factors and the repeated measurement throughout time in each maturation box was treated as a random effect to address the non-independence of samples. The best model was selected according to Akaike Information Criterion (AIC). Logarithmic and square root transformations of the data were necessary to ensure the normality and homogeneity of the variance of residuals of models. Student’s t tests were performed to determine the difference between compost. PLFAs data of each treatment were subjected to cluster analysis with the hclust function to determine the differences in the structure of the microbial community according to time. All statistical tests were evaluated at the 95% confidence level and values are given as the mean ± standard error.

## Results

### Temperature evolution

**Fig 1:**
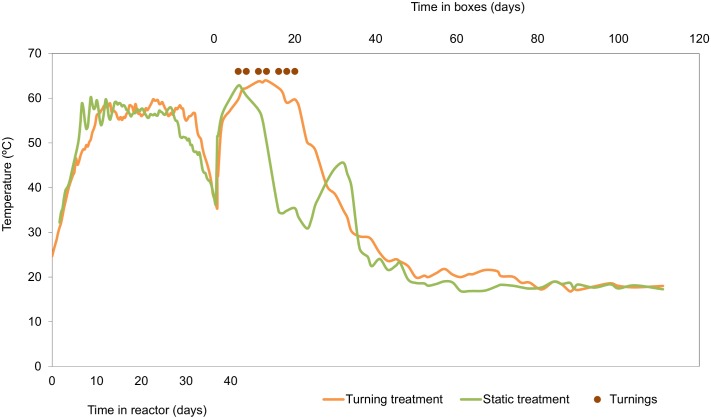
Temperature evolution during the reactor stage and the maturation stage for the turning treatment and static treatment.

The temperature profile during the reactor phase was similar in both tests, as can be seen in Fig 1; there were no significant differences between them (p > 0.05). The temperature slowly increased at the start of process, getting above 45°C starting on the 5th day. Both tests maintained thermophilic temperatures for 28 days. After emptying the reactors, reactivation of the process with thermophilic temperatures was quickly reached in the box tests.

The temperature evolutions of the two treatments during maturation were significantly different (p < 0.05). Static treatment saw temperatures maintained constantly in excess of 45°C for ten days, with a second peak temperature being registered on the 23rd day at no higher than 45°C; on the 14th day it was necessary to remoisten the material in order to keep moisture levels above 40%. Turning treatment saw the material in the boxes being turned seven times over a period of 20 days; the material was remoistened during three of these turns and thermophilic temperatures were maintained for 26 days. Both treatments saw oxygen levels superior to 14% during the material’s time in boxes.

### Composting parameters

**Fig 2:**
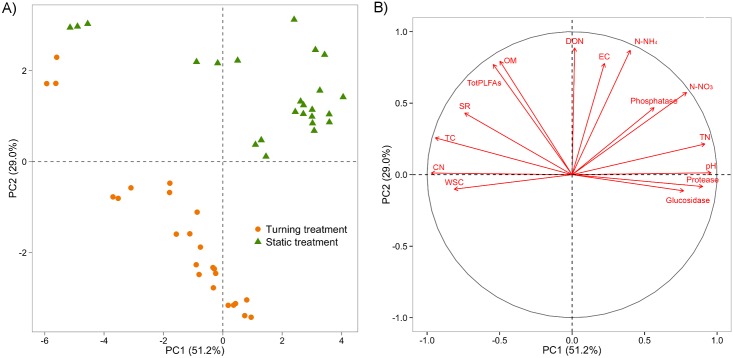
A) Principal component analysis (PCA) of the physico-chemical and biological variables of the turning treatment and static treatment B) Correlation circle showing the variables that define the components. CN: carbon-to-nitrogen ratio, DON: dissolved organic nitrogen, EC: electrical conductivity, OM: organic matter, SR: static respiration, TC: total carbon, TN: total nitrogen, totPLFAs: total amount of PLFAs, WSC: water soluble carbon.

The PCA was undertaken with the physico-chemical and biological variables shown in Fig 2. Three separate groups were yielded; they were the one formed at the start of the maturation phase of both tests, in other words when the materials left the reactor (time 0), the turning-treatment sample group, and the static-treatment sample group. The principal component 1 (PC1) accounted for 51.2% of the variance and significantly separated the three groups (F_2, 45_ = 337.95, p < 0.0001). The parameters responsible for the differences between the groups through PC1 were, fundamentally, pH (r = 0.96, p <0.0001), TC (r = -0.94, p <0.0001), TN (r = 0.92, p <0.0001), and the C/N ratio (r = -0.97, p <0.0001).

The parameters that contributed the most to separating the groups during the principal component 2 (PC2), which accounted for 29% of the variance, were DON (r = 0.88, p <0.0001), and ammonium (r = 0.90, p <0.0001); the three groups were significantly different from each other (F_2, 45_ = 29.09, p <0.0001). The initial material of the maturation stage contained high amounts of TC, OM, SR, and totPLFAs and low amounts of pH, TN, β-glucosidase and protease activity. Static treatment was characterized by higher levels of ammonium (p <0.0001), EC (p <0.0001), DON (p <0.0001), OM (p <0.0001), pH (p <0.0001), alkaline phosphatase (p <0.0001), protease (p <0.0001), and TN (p <0.0001) during the maturation process than those obtained via the turning treatment; static treatment produced lower levels of β-glucosidase (p <0.0001) than that achieved via turning. Organic-matter-loss at the end of maturation during the box phase was greater in the case of the turning treatment (42.5%) than the static one (35.6%); likewise, the percentage of carbon reduction was greater in the turned boxes (17.4%) than in the static ones (10.6%). Both treatments presented significantly different parameters in the middle of the process (56th day); these differences, with the exception of the C/N ratio, which was present at similar levels in both types of compost (Table 2), were maintained up to, and including, the final sampling.

**Table 2:** Compost characteristics after 56 and 112 days of maturation in the turning treatment and static treatment.

	56 days		112 days	
	Turning	Static	Turning	Static
**Organic Matter (%)**	74.3 ± 0.3	76.8± 0.8*	70.1 ± 0.5	75.7 ± 0.5*
**pH (mS cm**^**-1**^**)**	5.99 ± 0.03	6.61± 0.03*	6.01 ± 0.04	5.98 ± 0.05
**ratio C/N**	15.1 ± 0.5	12.7± 0.2*	13.8 ± 0.2	13.1 ± 0.2
**NH**_**4**_^**+**^**/NO**_**3**_^**-**^	0.17 ± 0.02	0.23 ± 0.01*	0.11 ± 0.01	0.21 ± 0.01*
**SR (mg O**_**2**_ **g**^**-1**^**OM h**^**-1**^**)**	0.48 ± 0.03	0.64 ± 0.02*	0.36 ± 0.02	0.47± 0.02*
**Sel-heating test**	-	-	clase V	clase V
**GI(%)**	82.7 ± 1.5	58.3 ± 2.1*	122.9± 6.9	77.3 ± 5.7*
**Fat (%)**	6.03 ± 0.03	9.14 ± 0.03*	4.21 ± 0.02	8.00 ± 0.01*

* indicates that samples for the same time between treatments are significantly different (Student t-test, p < 0.05) (SR: static respiration, GI: germination index).

### Microbial community

**Fig 3:**
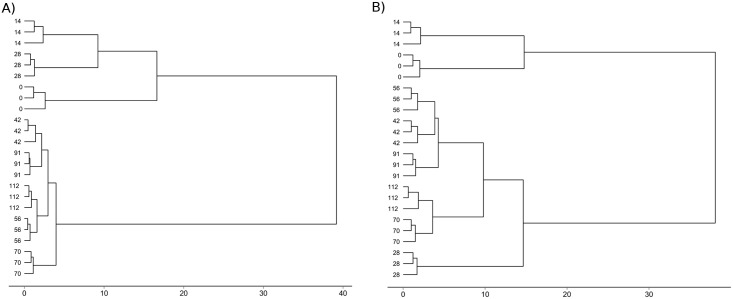
Dendrograms of cluster analysis based on PLFA profiles during the maturation stage in boxes of A) turning treatment and B) static treatment. Dendrograms were done with the data of 8 samplings taken during the maturation stage (day 0 to the 112th day) and were drawn based on Ward’s method on the Euclidean distance.

Cluster analysis based on PLFA profiles for turned boxes and static boxes show two clusters for each of the treatments. In the case of the turned boxes (Fig 3A), the samples on day 0, the 14th day, and the 28th day formed a cluster that was separate from the rest of the samplings, although the distance between day 0, the 14th day, and the 28th day was high; this suggests that the PLFA profiles were different. In the case of the second group (the 42nd day, 56th day, 72nd day, 91st day, and the 112th day), the similarities between the samples within the cluster were greater, with little distance between the samplings. With regards to static boxes (Fig 3B), the samples on day 0 and the 14th day formed a cluster, although the distance between one another was high; this suggests important differences between both samplings. Significant increases in bond distance were observed in the second cluster (the 28th day, 42nd day, 56th day, 70th day, 91st day, and the 112th day), indicating a low level of similarity between the samplings, especially between the 28th-day sampling and the rest, and between 70th-day-to-112th-day subgroup and the 56th-day-and-42nd-day-and-91st-day subgroup.

Table 3 shows the evolution of microbial groups during their time in boxes. The treatment performed on the material of the boxes significantly affected microbial-biomass evolution (p <0.0001), Gram + bacteria (p <0.0001), Gram—bacteria (p <0.0001), and fungi (p <0.0001). Likewise, significant differences caused by time (p <0.0001) and significant interaction between time and treatment (p <0.0001) were observed in the microbial biomass and all microbial groups. Both maturation treatments showed a high concentration of PLFA fungal biomarkers for the first samplings, with a decrease over time; this was especially true for the turning treatment (88.9% reduction in the case of turning versus 60.7% in the case of static treatment). Fungi were the main microbial group (averaging 48%) for practically the entire process, with the exception of the 28th-day-and-42nd-day-turning-treatment samplings in which Gram + bacteria were dominant. Gram + bacteria were reduced by 81% and 61% during the turning treatment and static treatment, respectively. The PLFA-Gram-negative-bacteria indicators showed a high increase on the 28th day of the static-treatment process; much higher levels than the turning treatment were maintained until the end of the process; static treatment showed an increase of 33%, whereas the turning treatment showed a 74% reduction. The compost produced by static treatment had a higher-level concentration of all the PLFA-group indicators than the turned compost.

**Table 3:** Changes in microbial groups: bacteria Gram +, bacteria Gram—and fungi and ratio of monounsaturated to saturated (mono/sat) PLFAs during the maturation stage of the turning treatment and static treatment.

Time (days)	Gram + (μg g^-1^dw)		Gram–(μg g^-1^dw)		Fungi (μg g^-1^dw)		mono/sat	
	Turning	Static	Turning	Static	Turning	Static	Turning	Static
**0**	139.9 ± 3.9	176.1 ± 8.9*	44.5 ± 4.1	39.0 ± 1.3	265.5 ± 2.1	276.4 ± 14.9	1.61 ± 0.10	1.25 ± 0.01*
**14**	119.8 ± 4.3	117.8 ± 5.7	20.0 ± 1.2	22.5 ± 1.4	244.9 ± 12.0	147.6 ± 10.3*	1.32 ± 0.07	0.99 ± 0.02*
**28**	122.4 ± 7.7	91.3 ± 1.9*	23.4 ± 1.6	90.6 ± 5.5*	109.5 ± 6.6	106.8 ± 8.5	1.04 ± 0.06	1.50 ± 0.09*
**42**	41.0 ± 5.9	57.5 ± 3.1	13.3 ± 0.8	52.8 ± 6.6*	29.8 ± 3.1	76.6 ± 8.3*	0.78 ± 0.06	1.22 ± 0.06*
**56**	26.4 ± 2.1	60.2 ± 3.9*	8.1 ± 1.0	52.9 ± 5.4*	27.4 ± 3.5	79.2 ± 3.8*	0.59 ± 0.09	1.27 ± 0.10*
**70**	39.4 ± 3.1	37.1 ± 1.9	10.4 ± 2.3	26.5 ± 3.8*	45.6 ± 4.9	76.3 ± 7.0*	0.78 ± 0.06	1.11 ± 0.06*
**91**	41.2 ± 4.2	61.4 ± 2.0*	14.5 ± 1.0	46.1 ± 4.3*	45.6 ± 2.3	94.7 ± 8.7*	0.83 ± 0.05	1.18 ± 0.05*
**112**	26.6 ± 2.0	68.5 ± 4.5*	11.7 ± 0.7	25.8 ± 0.4*	29.4 ± 0.6	108.7 ± 6.9*	0.79 ± 0.03	1.38 ± 0.06*

* indicates that samples for the same time between treatments are significantly different (Student t-test, p < 0.05) dw: dry weight

The mono/sat ratio achieved by static treatment was greater than that achieved by turning starting from the 28th day, the latter maintaining stable levels starting from the 70th day.

## Discussion

The temperature profiles of both tests while in the reactor, corresponding to the intensive stage, showed patterns typical of the composting process; in other words, temperatures increasing to thermophilic levels followed by maintaining said temperature and a subsequent decline in temperature until reaching mesophilic levels. Despite thermophilic conditions being reached slowly, compost hygienization was ensured by continuously maintaining temperatures above 55°C for more than 15 days [27]. Similar temperature profiles have been observed by other authors during the composting of fatty wastes [8,28,29], therefore, a high level of fat causes the compost to reach thermophilic temperatures more slowly but once reached they cause the compost to maintain thermophilic temperatures for longer due to the fact that the lipids present provide a greater amount of energy than other organic compounds. The sludge used in the research came from the purification of wastewater generated by the production of precooked and frozen foods, such as breaded and battered fish and cephalopods, so the lipid content of the waste was high (Table 2). Thus, the sludge used had an optimum amount of fats for microbial degradation through composting since the appropriate values for this process should not exceed 20–25% according to studies carried out by Fernandes et al. [28] for urban, agricultural and industrial waste. Likewise, the low pH of the sludge, with values less than 5, was able to inhibit thermophilic microorganisms and slow the transition from mesophilic to thermophilic temperatures during the initial composting reactor stage, as observed by Sundberg et al. [30]. The lack of difference between the temperature profiles of both tests in the reactor makes it possible to carry out statistical analysis conducive to checking the current study’s objectives by submitting the material to the same processing conditions during the intensive phase.

After the static-reactor stage, the pre-composted material was homogenized and placed in boxes, resulting in a quick reactivation for both treatments, indicating that the material had enough biodegradable substrates to allow it to self-heat until it reached thermophilic temperatures. Likewise, turnings carried out during the first several days of the material being in the boxes facilitated the extension of thermophilic conditions more so than the static approach. Therefore, turning reactivated the process of incorporating the least-degraded material and, as a result, made it possible to supply easily assimilable substrates to the microbial biomass, starting from the exterior of the boxes and moving inwards. Ruggieri et al. [8] have suggested that turning during the composting of fatty wastes is preferable to the static system because it prevents the mixture from forming clumps and compacting. Likewise, Albuquerque et al. [31] have observed that, during alperujo composting, forced-air ventilation is only effective when done along with turning because it improves the substrate’s porosity. It has also showed that turning, after static-reactor composting seafood sludge with forced ventilation, makes lengthening the thermophilic phase possible. At most composting facilities, turnings of the pre-composted waste in the maturation stage are not based on the biological process, but on industrial criteria (increase space, move the material to another location, dry the material…) so, the implementation of protocols for the maturation stage is considered important to improve the composting process as has been observed.

According to multivariate analysis, the treatment applied while the material is in the boxes determines its physico-chemical, and biological, development vis-à-vis compost stabilization and maturation. Turning the material during the maturation stage allows for compost to degrade to a greater degree, suggesting that the time needed to achieve stability is reduced when controlling the process is done under dynamic conditions. Other authors have found similar results with regards to all stages of the composting process and not only the maturation stage in particular. For example, Brito et al. [32] have found that turning increases the degradation rate during the composting of cattle slurry; statically-treated piles require more processing time to reach organic-material values similar to that obtained in compost that had been turned. Nikaeen et al. [33] have also pointed out that the time needed to stabilize organic compounds in static piles is greater due to the lack of heat exchange. Furthermore, it has been stated that turning and how often it occurs affects the EC, pH, TC, TN, C/N ratio, GI, and temperature during the composting of different types of waste products [9–12,34]. Similar results have been obtained from this study, showing that turning pre-composted waste in line with temperature criteria following static-reactor composting affected the pH, electrical conductivity, ammoniacal nitrogen, dissolved organic nitrogen, organic matter, enzymatic activities, total nitrogen and total carbon; this led to the compost becoming stable in less time than through static conditions for the maturation stage. Turning-treatment samples starting from the 56th day showed stable conditions; some parameters, like the respiration rate and the germination index for example, were improved by prolonging the process. Nonetheless, the statically-treated compost had insufficient quality parameters on the 56th day; there were also significant variations throughout the entire process. The analysis of the compost after 112 days in the maturation boxes showed parameters that indicated stability in both cases; these parameters included the maximum rating for the Dewar self-heating test (class V, mature compost) [35], and a respiration rate that was less than 0.5 mgO_2_ g^-1^SV h^-1^ [36]. Also, both types of treatment produced compost that reached optimum C/N-ratio levels of less than 20 [35]. However, the turned compost had an ammonium/nitrate ratio of less than 0.16 [37] and a germination index superior to 80% [38], which showed a greater degree of maturity than the compost that received the static treatment. Controlling through turning significantly affected physico-chemical, and biological, characteristics as well as microbial activity; making it possible to achieve a more stable, and more mature, product in less time than the unhandled material. So, when a composting facility is decided to work with a static technique for the intensive stage, the control of the maturation by turnings could optimize the process to obtain a compost of better quality in less time.

It is known that the nature of organic substrates and temperature are the principal factors that determine the composting process with respect to microbial dynamics [4,39,40]. Due to the fact that the initial material placed in boxes after having been composted in the reactor were very similar on a physico-chemical, and biological, level -as observed using multivariate analysis- the changes in the microbial community’s structure were mainly produced as a consequence of the temperature reached in the boxes during the first weeks of maturation stage. Bacterial and fungal populations decreased with both treatments, which is an expected decline as the most easily assimilable substrates are consumed, reducing the available food for microbial growth. Nevertheless, turning produced a less noticeable decrease of Gram + bacteria during the first few weeks, which was due to Gram + bacteria, in particular those that belong to the genus *Bacillus*, dominating during the thermophilic phase of composting [4] and turning boxes maintaining thermophilic temperatures for more days. In the same way, it is known that increasing temperatures caused fungi to decrease [4,41], although in the case of turning the population stayed the same for the first month when the temperatures were high and this could mean that were thermophilic or thermo-tolerant fungi present [13,42]. Choi and Park [43] have also observed high activity of yeast in thermophilic composting of food waste, indicating that the ability of yeast to grow at a lower pH than bacteria could explain their presence. The pH and the lipid content of the pre-composted sludge allowed for the development of thermophilic fungi and turnings allowed for the input of food to initially maintain their population. So, the cluster obtained by analyzing the PLFAs found in the first two static-treatment samplings, and those obtained from the first three turning-treatment samplings, are mainly characterized by the temperature reached during the first few weeks which in turn depended on the treatment carried out.

It is normal for the number of bacteria to lower in population, but increase in terms of diversity, and for fungi to increase both in terms of quantity and diversity during the maturation phase itself [4]. Nonetheless, a decrease in temperature did not produce an observable increase in how abundant fungi were; both treatments, however, were characterized by the fungal biomass being predominant, particularly in the case of static treatment, throughout the entire process. Amir et al. [44] found that fungi are more present in wastes with a high level of fatty acids. Likewise, Villar et al. [13] suggested that the influence the initial material, in this case lipid in nature and pH, has on the maturation phase is reflected by microbial diversity; this means that although the waste undergoes significant degradation during the composting process, the properties of the starting material determine the dominant microbial groups throughout the maturation stage. Following the high-temperature phase, there was a large increase in Gram-negative-bacteria PLFA biomarkers with respect to static treatment. Due to Gram—bacteria’s limited resistance to temperature the normal course of action is for thermophilic Gram+ bacteria to give way to mesophilic Gram—bacteria during composting or maturation [45]. It has been confirmed that bacteria belonging to the phylum *Bacteroidetes* and class *Alphaproteobacteria* frequently dominate both in compost after it has reached high temperatures and in compost that has not matured [46–48]. Likewise, other Gram—bacteria like *Gammaproteobacteria* are dominant in cured compost [48]. However, the fluctuation of Gram—bacteria together with the physico-chemical data could indicate a lack of stability in the static treatment in the final stages of maturation. On the contrary, all the microbial groups in the turned compost stayed at similar levels following the high-temperature phase; this produced microbial-community homogeneity as observed in group II of the multivariate analysis. Microbial-population values in turned compost stayed low once it had reached stability and maturation parameters, whereas the increase in Gram + bacteria and fungi obtained from static treatment could indicate the availability of biodegradable substrates [49]. Turning the material made it possible to maintain more stable and more similar microbial diversity after reaching mesophilic temperatures whereas not homogenizing the boxes maintained a greater degree of difference between samplings. Cahyani et al. [46] have pointed out that the microbial community remained stable during the maturation phase of composting rice straw. On the other hand, Danon et al. [48] observed changes to the microbial communities during the year-long composting of biosolids. These authors observed that in the case of bacterial populations specialized in breaking down macromolecules such as lignocellulose, stability was maintained during the intermediate part of the maturation phase.

Likewise, the mono/sat ratio values after high temperatures in the static treatment indicated that microbial communities do not have limited resources and there are enough nutrients for their growth. However, in the compost that received the turning treatment the mono/sat ratio showed that the microbial communities were subjected to nutritional and physiological stress. Jindo et al. [50] have suggested that low mono/sat ratio numbers demonstrates the final part of organic material’s transformation into compost and Bastida et al. [51] have pointed to a greater degree of carbohydrate-substrate biodegradability when the numbers of the aforementioned ratio are high. Therefore, mono/sat-ratio stabilization can be indicative of mature compost. Bacterial-population stabilization, as a result of running out of easily degradable substrates, can indicate the degradation of more calcitrant compounds, ergo turning the material while it is in maturation stage allows for a greater degree of organic-material degradation and microbial succession towards a more stable mesophilic community, reflecting compost maturation.

## Conclusions

The fresh-compost-turning system made it possible to maintain temperatures and, as a result, prolonged the thermophilic phase; this allowed for a high level of organic-matter degradation over a longer period. Controlling the composting of highly-energy- waste through turning after the most intensive stage in the reactor made it possible to achieve stability and maturity in a shorter time frame than statically treating pre-composted waste. Studying microbial dynamism has helped to characterize the state of degradation of organic matter as stable compost showing a stable microbial structure whereas the presence of biodegradable substrates causes significant changes to microbial populations. Looking at the results, composting plants that utilize turning during the maturation period optimize the process by cutting the time needed to reach sufficiently high levels of quality.
